# Chronic wounds in Sierra Leone: pathogen spectrum and antimicrobial susceptibility

**DOI:** 10.1007/s15010-022-01762-6

**Published:** 2022-02-23

**Authors:** Frieder Schaumburg, Jonathan Vas Nunes, Giulia Mönnink, Abdul-Mac Falama, James Bangura, Hanna Mathéron, Amara Conteh, Maxwell Sesay, Aminata Sesay, Martin P. Grobusch

**Affiliations:** 1 Institute of Medical Microbiology, University Hospital Münster, Domagkstraße 10, 48149 Münster, Germany; 2 Masanga Medical Research Unit (MMRU), Masanga, Sierra Leone; 3 Department of Infectious Diseases, Center of Tropical Medicine and Travel Medicine, Amsterdam University Medical Centers, Amsterdam, The Netherlands; 4 Ministry of Health and Sanitation, Freetown, Sierra Leone

**Keywords:** Wound Infection, Africa, Staphylococcus aureus, Pseudomonas aeruginosa, Antimicrobial resistance

## Abstract

**Purpose:**

Chronic wounds are frequently caused by, or super-infected with, a broad spectrum of bacteria. To guide treatment, healthcare providers need to know the bacterial spectrum and antimicrobial resistance rates to be anticipated. As these data are largely missing for Sierra Leone, we performed a microbiological study on chronic wound infections.

**Methods:**

Wound swabs were analysed for bacteria using culture-based methods. Antimicrobial susceptibility testing was done with Vitek2® automated system and EUCAST clinical breakpoints. Selected resistance phenotypes were confirmed by molecular methods (e.g. *mecA/C*) and genotyping.

**Results:**

Of 163 included patients, 156 (95.7%) had a positive wound culture. *Pseudomonas aeruginosa* (*n* = 75), *Klebsiella pneumoniae* (*n* = 42), *Proteus mirabilis* (*n* = 31), *Staphylococcus aureus*-related complex (*n* = 31) were predominant. Among Gram-negative rods, resistance rates were high for piperacillin/tazobactam (3–67%), cefotaxime (19–71%), and ciprofloxacin (13–60%). Among isolates of the *S. aureus*-related complex, 55% were methicillin resistant (CC8, PVL-negative).

**Conclusion:**

The high antimicrobial resistance rates in bacteria from chronic wounds strongly speaks against the use of empirical systemic antibiotic therapy if patients do not show signs of systemic infections, and supports the strategy of local wound care.

## Introduction

One of the current and future challenges in medicine is the ongoing pandemic of antimicrobial resistance. To stem the tide of antimicrobial resistance, numerous bundles of measures are suggested, such as infection control and prevention, access to clean water and sanitation, surveillance, rapid diagnostics, vaccines and, probably most importantly, ‘improvement of antimicrobial consumption in humans and animals’ [1]. To improve antimicrobial use, local data on antimicrobial resistance need to be available to prescribers to make an informed decision on the selection of antimicrobial agents, and to set up treatment guidelines. However, our hands are tied, if these data are non-existent, which is the case in many resource-limited settings without microbiology facilities [2].

Reports from West Africa (e.g. Benin, Côte d'Ivoire, Ghana, Sierra Leone, Togo) provide an impression of the extent of antibiotic resistance in human infections: extended-spectrum beta-lactamase (ESBL)-producers are common in *Escherichia coli* (36%) and *Klebsiella pneumoniae* (48–84%); methicillin-resistance is widespread among *Staphylococcus aureus* (MRSA, 14.3–34.6%) [3–5]. In Nigeria, carbapenemases (*bla*_NDM_, *bla*_OXA-181_) in ESBL-producing Enterobacterales (27.4%) demonstrate futility of a de-escalation strategy in antibiotic selection, not to mention that third-line drugs (e.g. colistin, ceftazidime/avibactam) are not even available [6]. The high proportion of WHO priority pathogens (carbapenem-resistant, ESBL-producing Enterobacterales, MRSA) warrants further investigation in the West African region. We therefore analysed the bacterial spectrum of chronic wound infections and antimicrobial susceptibility in a district hospital in Sierra Leone.

## Methods

### Ethics

The study protocol was endorsed by the Scientific Review Committee (SRC) of the Masanga Medical Research Unit (MMRU). Ethical approval was obtained by the National Ethics committee of Sierra Leone, the Sierra Leone Ethics and Scientific Review Committee (approval granted on 25th January 2019). All participants signed a written informed consent prior to inclusion.

### Study setting

Masanga Hospital is a secondary-level-of care district hospital in rural Tonkolili district, Sierra Leone. As a national referral centre for wound care, many patients are seen for their wounds, burns, amputations and skin grafts. Basic haematology and biochemistry laboratory tests are available, as well as sickle cell electrophoresis, basic X-ray modalities, rapid testing for HIV, hepatitis B and C, and microscopy for leprosy, Ziehl–Neelsen- and Gram-staining. Bacteriological testing is not available in Sierra Leone, except for tuberculosis confirmation testing in the National TB referral centre in Freetown.

### Study population

Demographic, clinical, laboratory and diagnostic data on all patients presenting at Masanga Hospital with a wound were collected as part of a larger hospital based-registry. All patients with a wound or with multiple wounds seen at the Masanga Hospital out-patient department or admitted to Masanga Hospital between 10-07-2019 and 30-11-2020 were included in this study. Patients were included regardless of aetiology of the wound (e.g. wound originating from trauma, infection, burn or drug reaction). Exclusion criteria were: refusal of informed consent for the study, closed wounds (e.g. from blunt trauma, haematomes) and surgical site infections. Patients who only refused voluntary HIV-testing were not excluded from this study to prevent any form of stigmatisation.

### Microbiology

After removing any potential wound coverage such as bandages, traditional leaves or necrotic skin with a dry sterile gauze or sterile forceps, one swab per patient was taken by swabbing thoroughly both the central areas and the (undermined) borders of the wound. The swabs were returned in their sterile covering in a sterile fashion.

After sampling, swabs (Transswab, MWE, Corsham, England) were stored in Amies transport medium at 2–7 °C and were shipped at room temperature to Germany for further analysis. The swabs were cultured for 18–24 h on Columbia blood-, chocolate-, McConkey- and CN (colistin-nalidixic acid) agar (all BD, Heidelberg, Germany) at 35 °C, ambient air. Anaerobic culture was done on Schaedler-KV agar (Oxoid, Wesel, Germany) for 48 h (35 °C). The Institute of Medical Microbiology, University of Münster regularly participates in external quality assessments for species identification and susceptibility testing and has a quality management system.

Species identification was done by MALDI-TOF mass spectrometry (Microflex Bruker, Bremen, Germany) using the MBT Compass software (version 4.1.80, Bruker). As this method cannot delineate *Streptococcus canis*, *Streptococcus dysgalactiae* and *Streptococcus pyogenes* from each other, we combine these species into “β-hemolytic streptococci” in this work. Similarly, *S. aureus* and *S. argenteus* were combined to “*S. aureus*-related complex” [7].

Antimicrobial susceptibility testing (AST) was done with Vitek2 automated systems (bioMérieux, Marcy l’Étoile, France). We used the following standard set of AST cards: AST-N214 (*Enterobacterales, Acinetobacter* spp.), AST-N248, -N389 (*Pseudomonas* spp.*, Alcaligenes* spp.), AST-P654 (*Staphylococcus* spp*.*) and AST-P655 and AST-ST03 (*Streptococcus* spp*.*). Chloramphenicol is commonly used in the study area but absent in the majority of test panels. Susceptibility to chloramphenicol was, therefore, tested using disk diffusion test (30 µg, Oxoid). All AST were performed according to EUCAST, and results were interpreted post hoc according to EUCAST clinical breakpoints (version 11, 2021) [8]. Isolates categorised as ‘susceptible, increased exposure’ were grouped together with susceptible isolates.

### Molecular analyses

*S. aureus*-related complex isolates were subjected to *spa* typing [9] and multilocus sequence typing (MLST) [10]. In addition, these isolates were screened for Panton-Valentine leukocidin (PVL) and *mecA*/*mecC* using a commercial kit (eazyplex® MRSAplus, Amplex, Gars-Bahnhof, Germany) to test whether the hypervirulent USA300 methicillin-resistant *S. aureus* (ST8 MRSA, PVL-positive, t008 or related) clone is circulating in the study area. This clone was sporadically detected in some sub-Saharan countries (e.g. Côte d’Ivoire, Gabon) [11]. Clonal complexes (CC) were obtained from the ‘Public databases for molecular typing and microbial genome diversity’ (https://pubmlst.org/organisms/staphylococcus-aureus) or deduced from the MLST allelic profiles using BURST.

Enterobacterales with reduced susceptibility to carbapenems (resistant, susceptible increased exposure) were screened for *bla*_NDM_, *bla*_KPC_, *bla*_OXA-48_, *bla*_OXA-181_ and *bla*_VIM_ (eazyplex® SuperBug CRE, Amplex). Colistin-resistant Gram-negative rods were tested for *mcr-1* (eazyplex® SuperBug mcr-1, Amplex).

### Statistics

Only species/genera with ≥20 isolates and only one isolate of each species from individual patients were entered in the analysis of resistance rates.

An association of co-detected species was tested using the McNemar's test with the continuity correction as implemented in “R” (significance level: 0.05).

## Results

**Fig. 1:**
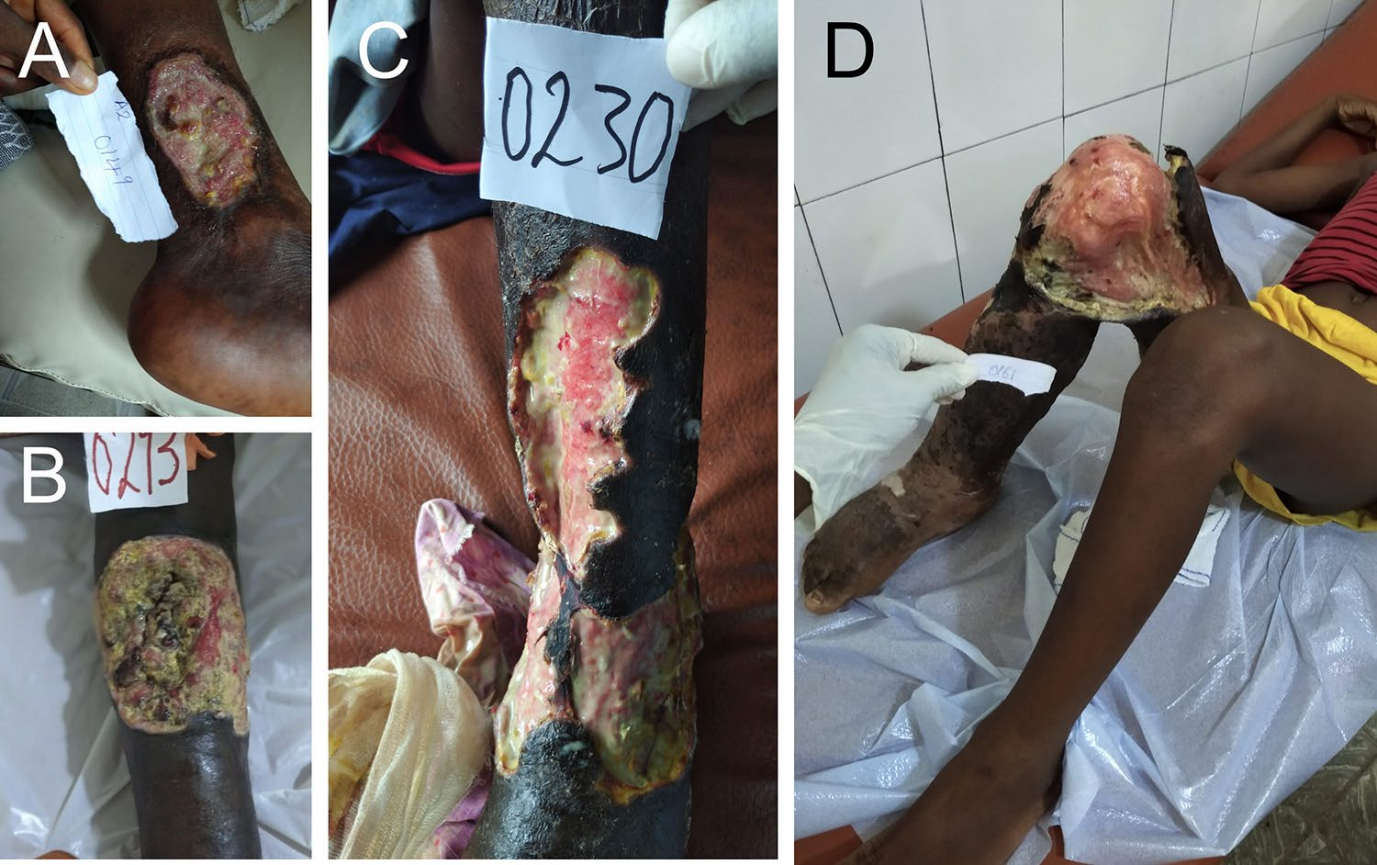
Chronic wounds in Sierra Leone. The majority of wound are located at the legs (**A**–**D**). Some of them have an undermined edges (**C**, **D**). Mixed bacterial flora was common

In total, 163 patients were included (11% females, *n* = 18). The median age (range) was 40 years (0–88). Wounds were located at the foot (*n* = 56), lower leg (*n* = 79), thigh (*n* = 5), arm (*n* = 2) and other areas (*n* = 21). Figure 1 shows examples of wounds and skin ulcers. The median wound diameter was 10 cm. According to treating clinicians, the majority of wounds were superficial [84% (*n* = 137) subcutaneous tissue visible] with deep edges (53%, *n* = 86) and presence of slough (60%, *n* = 98). In some of the patients the clinicians described signs of acute infection of the skin (29%, *n* = 47) or bone (10%, *n* = 16) or an offensive odour (30%, *n* = 49). A minority of wounds showed sign of granulation (36%, *n* = 59) at first presentation.

None of the patients were under antibiotic treatment prescribed by registered clinicians at the time of sampling (incl. 14 days prior to sampling). A majority of patients (50%, *n* = 82), however, used at least one (28%, *n* = 46), two (20%, *n* = 33) or three (2%, *n* = 3) different systemic antibiotics of unknown dosage, duration and quality purchased over-the-counter in local pharmacies at some time prior to arriving in Masanga. In these patients (*n* = 82), the predominant prescribed antibiotics were ampicilline/cloxacilline (Ampiclox®, 84%, *n* = 69), ceftriaxone (24%, *n* = 20) and ampicilline (13%, *n* = 11).

In addition to traditional topical treatments such as iodine, sugar and salt, 61 patients (37%) used a topical antibiotic purchased over the counter prior to presenting at the hospital. The majority of patients (55%, *n* = 82) reported having seen a traditional healer prior to presenting at Masanga Hospital.

The median duration of the wounds was 12 months (range 0.23–36). The aetiology of wounds was trauma (12%, *n* = 19), abscess (73%, *n* = 119) or unknown (14%, *n* = 22).

Of all patient included, 156 (95.7%) had a positive culture from wound swabs with a median of three (range 1–7) different species. A total of 461 isolates belonging to 60 different species were detected; among the 163 wound swabs, the majority yielded *Pseudomonas aeruginosa* (46%, *n* = 75), *K. pneumoniae* (26%, *n* = 42), *Proteus mirabilis* (19%, *n* = 31), *S. aureus* related complex (19%, *n* = 31), *Morganella morganii* (15%, *n* = 25), *Alcaligenes faecalis* (14%, *n* = 22) and β-haemolytic *streptococci* (12%, *n* = 20). Fungi (6%, *n* = 9) were less frequently detected and were identified as *Candida tropicalis* (4%, *n* = 6), *Candida orthopsilosis* (1%, *n* = 2) and *Candida krusei* (1%, *n* = 1). Noteworthy, in patients with an *S. aureus* infection (*n* = 31), *P. aeruginosa* was co-detected in 13 cases (42%). The association between *S. aureus* and *P. aeruginosa* was significant (*p* = 0.001, McNemar test).

Antimicrobial resistance rates of gram-negative bacteria were favourable (e.g. <20%) for *Proteus* spp. (piperacillin/tazobactam, cephalosporins, fluoroquinolones*), M. morganii* (piperacillin/tazobactam, ceftazidime) and *Citrobacter* spp. (cephalosporins, Table 1). The other species yielded high resistance rates, for instance, against piperacillin/tazobactam (24–67%), cefotaxime (26–71%), and ciprofloxacin (37–60%).

**Table 1:** Antimicrobial susceptibility of gram-negative bacteria from wound infections

	Antimicrobial resistance [%, (*n*)]												
Species (*n*)	Ampicillin	Ampicillin/sulbactam	Piperacillin/tazobactam	Cefuroxime^a^	Cefotaxime	Ceftazidime	Ertapenem	Meropenem	Gentamicin	Ciprofloxacin	Trimethoprim/sulfamethoxazole	Chloramphenicol	Colistin
*Citrobacter* sp. (21)^b^	100% (21)	71% (15)	24% (5)	NA	19% (4)	19% (4)	0% (0)	0% (0)	38% (8)	38% (8)	48% (10)	38% (8)	ND
*Klebsiella* sp. (55)^c^	100% (55)	78% (43)	51% (28)	56% (31)	56% (31)	53% (29)	0% (0)	0% (0)	58% (32)	47% (26)	67% (37)	46% (25)	ND
*Klebsiella pneumoniae* (42)	100% (42)	83% (35)	67% (28)	71% (30)	71% (30)	67% (28)	0% (0)	0% (0)	74% (31)	60% (25)	83% (35)	55% (23)	ND
*Morganella morganii* (25)	100% (25)	96% (24)	12% (3)	NA	40% (10)	16% (4)	4% (1)	0% (0)	48% (12)	60% (15)	80% (20)	80% (20)	NA
*Proteus* sp. (53)^c^	77% (41)	28% (15)	6% (3)	NA	19% (10)	9% (5)	4% (2)	2% (1)	21% (11)	13% (7)	47% (25)	40% (21)	NA
*Proteus mirabilis* (31)	61% (19)	32% (10)	3% (1)	26% (8)	26% (8)	13% (4)	3% (1)	0% (0)	32% (10)	19% (6)	65% (20)	48% (15)	NA
*Pseudomonas aeruginosa* (75)	NA	NA	32% (24)	NA	NA	24% (18)	NA	0% (0)	56% (19)	37% (28)	NA	NA	1% (1)

NB: *Alcaligenes faecalis* (*n* = 22) were not tested as no EUCAST clinical breakpoints are currently available; *NA *not applicable, *ND* not done

^a^For intravenous application

^b^Including *Citrobacter amalonaticus* (*n* = 1), *Citrobacter freundii* (*n* = 13), *Citrobacter koseri* (*n* = 7)

^c^Including *Klebsiella aerogenes* (*n* = 6), *Klebsiella oxytoca* (*n* = 4), *Klebsiella pneumoniae* (*n* = 42), *Klebsiella* sp. (*n* = 1), *Klebsiella variicola* (*n* = 2)

^c^Including *Proteus hauseri* (*n* = 14), *Proteus mirabilis* (*n* = 31), *Proteus penneri* (*n* = 1), *Proteus vulgaris* (*n* = 7)

No carbapenemases (*bla*_NDM_, *bla*_KPC_, *bla*_OXA-48_, *bla*_OXA-181_ and *bla*_VIM_) were detected in carbapenem-resistant *M. morganii* (*n* = 1) and *Proteus* spp. (*n* = 3). The colistin resistant *P. aeruginosa* was negative for *mcr-1*.

For isolates belonging to the *S. aureus*-related complex (*n* = 31), resistance rates for standard drugs were high (penicillin 97%, oxacillin 55%; Table 2). Chloramphenicol was the only agent with resistance rates <20% that is available locally and that can be administered orally (Table 2). All isolates were susceptible to glycopeptides, linezolid and daptomycin. In total, 17 methicillin-resistant *S. aureus*/*argenteus* (MRSA) were detected (*mecA* positive; Table 3).

**Table 2:** Antimicrobial susceptibility of gram-positive cocci from wound infections

	Antimicrobial resistance [%, (*n*)]											
Species (*n*)	Penicillin	Oxacillin	Gentamicin	Levofloxacin	Clindamycin	Erythromycin	Tetracycline	Trimethoprim/sulfamethoxazole	Vancomycin	Linezolid	Daptomycin	Chloramphenicol
*S. aureus* related complex (31)^a^	97% (30)	55% (17)	36% (11)	48% (15)	23% (7)	45% (14)	74% (23)	77% (24)	0% (0)	0% (0)	0% (0)	19% (6)
*β-hemolytic streptococci* (20)^b^	0% (20)	NA	NA	0% (20)	35% (7)	35% (7)	100% (20)	25% (5)	0% (0)	0% (0)	ND	30% (6)

NB: *Alcaligenes faecalis* (*n* = 22) were not tested as no EUCAST clinical breakpoints are currently available; *NA* not applicable, *ND* not done

^a^Including *Staphylococcus aureus* (*n* = 30), *Staphylococcus argenteus* (*n* = 1)

^b^Including *Streptococcus dysgalactiae* (*n* = 19) and *Streptococcus pyogenes* (*n* = 1)

**Table 3:** Population structure of *Staphylococcus aureus*-related complex from wound infection, Sierra Leone

*mecA*	Cloncal complex	MLST sequence type	*spa* type (*n*)	Panton-Valentine leukocidin
Negative	CC1	ST1	t19856 (1)	Positive
	CC5	ST5	t311 (3)	Negative
		ST6662	t062 (1)	Positive
	CC8	ST8	t1476 (2)	Negative
	CC15	ST15	t084 (2)	Negative
			t084 (1)	Positive
			t1494 (1)	Positive
	CC152	ST152	t355 (2)	Positive
	NT	NT	NT (1)	Positive
Positive	CC5	ST5	t442 (3), t9798 (1)	Negative
	CC8	ST8	t1476 (2), t064 (2), t451 (2), t054 (1)	Negative
		ST672	t3841 (1)	Negative
		ST789	t091 (1)	Negative
		ST6194	t1476 (1)	Negative
	CC2196	ST6454	t1476 (1)	Negative
		ST6455	t1476 (1)	Negative
	CC2250	ST2250	t6675 (1)	Negative

*NT* non-typeable

Of all *S. aureus* (*n* = 31), the Panton-Valentine leukocidin was detected in 23% (*n* = 7); all MRSA were PVL-negative (Table 3). The dominant PVL-positive clonal complexes (CC) were CC1, CC15 and CC152. CC8 was the dominant MRSA clonal complex. In terms of CCs, methicillin susceptible *S. aureus*/*argenteus* were more diverse (5 CCs/14 isolates) compared to MRSA (3 CCs/17 isolates). The *S. argenteus* (*mecA* positive, PVL negative) belonged to ST2250 (*spa* type t6675).

## Discussion

In this study, we observed high rates of antimicrobial resistance among Gram-negative bacteria and *S. aureus*-related complex from wound infections.

Differentiation between bacterial, parasitic, lymphatic or traumatic origin of wounds is often difficult, and bacterial superinfection in presumably high. Therefore, the question whether the isolates were causative for the wounds, or represented colonisers, cannot be resolved. Wounds can have various causes and contributing factors, some of which are also widespread in the study area (e.g. microangiopathy, sickle cell disease, malnutrition). In addition, we might have missed causative pathogens that are difficult/impossible to culture (*Mycobacterium leprae*, *Treponema pallidum pertenue*, *Blastomyces*, *Coccidioides*). We believe that Buruli ulcer (BU) caused by *Mycobacterium ulcerans* is most likely a main driver of wounds in our study region as Sierra Leone is endemic for BU and the majority of lesions were located at the lower legs. The lesions often had undermined borders (Fig. 1C, D), and evolved from nodules that might have been misinterpreted as abscesses [12].

The bacterial spectrum in our study (Tables 1, 2) corresponds to what is expected in chronic wounds, including BU with a predominance of *P. aeruginosa*, *Klebsiella* sp., *Proteus* spp. and *S. aureus* [13–15]. The high rates of resistance against piperacillin/tazobactam particularly against *P. aeruginosa* (32%) and *K. pneumoniae* (67%) appears surprising, as this drug is not used in the study region. However, CTX-M 15 (associated with ceftriaxone resistance) is widespread among ESBL-producers in sub-Saharan Africa and often co-located with OXA-1 on the same plasmid [16]. OXA-1 confers resistance to piperacillin/tazobactam and might explain the high resistance rates by a co-selection of CTX-M 15 [17].

The proportion of MRSA among *S. aureus*-related complex was high (55%; Table 2) and exceeds by far previous reports of chronic wounds from West Africa (13–38%) [13, 14]. The predominance of MRSA CC8 (PVL negative; Table 3) reflects the clonal structure of MRSA in West Africa and could point toward the expansion of this lineage [18].

In general, the high resistance rates found in both gram-negative rods and gram-positive cocci strongly argues against any empirical use of antimicrobial agents in the management of patients without systemic signs of infection, as the medical benefit is low/absent. Instead, local antiseptics (e.g. povidone, vinegar and honey) and improved wound care are most likely more effective [19].

Our work has the following limitations: first, the long timespan between sampling and culture is not optimal for fastidious species. However, the impact on the bacterial spectrum is most likely little, as the major common species of skin and soft tissue infections were found. However, we might have missed species due to overgrowth [20]. Second, we were unable to assess the bacterial concentration in wound fluids. Concentrations of >10^6^ colony-forming units/ml are considered to be associated with infection and subsequent healing delay [13]. Third, patients treated at the wound clinics might be a selected population with an overrepresentation of severe cases that could not have been handled in referring hospitals/health care institutions.

## Conclusion

The high antimicrobial resistance rates in bacteria colonizing/infecting chronic wounds strongly argue against the use of empirical therapy if patients do not show signs of systemic infections and supports the strategy of improved wound care. Sustainable capacity building to perform microbiological analyses locally is the most essential point to approach the pandemic of antimicrobial resistance in low and middle-income countries. This should go along with training programs on the prescription of antimicrobial agents and a restriction of over-the-counter sale without prescriptions.
